# Redox control and autoxidation of class 1, 2 and 3 phytoglobins from *Arabidopsis thaliana*

**DOI:** 10.1038/s41598-018-31922-4

**Published:** 2018-09-12

**Authors:** Augustin C. Mot, Cristina Puscas, Patricia Miclea, Galaba Naumova-Letia, Sorin Dorneanu, Dorina Podar, Nico Dissmeyer, Radu Silaghi-Dumitrescu

**Affiliations:** 1 Research Center for Advanced Chemical Analysis, Instrumentation and Chemometrics, Babes-Bolyai University, 11 Arany Janos Street, RO-400028 Cluj-Napoca, Romania; 2 Faculty of Chemistry and Chemical Engineering, Babes-Bolyai University, 1 Mihail Kogalniceanu Street, RO-400084 Cluj-Napoca, Romania; 3 Independent Junior Research Group on Protein Recognition and Degradation, Leibniz Institute of Plant Biochemistry, Weinberg 3, D-06120 Halle (Saale), Germany; 4 Faculty of Biology and Geology, Babes-Bolyai University, 1 Mihail Kogalniceanu Street, RO-400084 Cluj-Napoca, Romania

## Abstract

Despite a recent increase in interest towards phytoglobins and their importance in plants, much is still unknown regarding their biochemical/biophysical properties and physiological roles. The present study presents data on three recombinant *Arabidopsis* phytoglobins in terms of their UV-vis and Raman spectroscopic characteristics, redox state control, redox potentials and autoxidation rates. The latter are strongly influenced by pH for all three hemoglobins – (with a fundamental involvement of the distal histidine), as well as by added anion concentrations – suggesting either a process dominated by nucleophilic displacement of superoxide for AtHb2 or an inhibitory effect for AtHb1 and AtHb3. Reducing agents, such as ascorbate and glutathione, are found to either enhance– (presumably via direct electron transfer or via allosteric regulation) or prevent autoxidation. HbFe^3+^ reduction was possible in the presence of high (presumably not physiologically relevant) concentrations of NADH, glutathione and ascorbate, with differing behaviors for the three globins. The iron coordination sphere is found to affect the autoxidation, redox state interconversion and redox potentials in these three phytoglobins.

## Introduction

Hemoglobins (Hb) are globular proteins that use heme as cofactor and are able to reversibly bind molecular oxygen (O_2_). However, the fact that Hb can also bind ligands such as nitric oxide (NO), carbon monoxide (CO) and hydrogen sulfide (H_2_S), or even other small organic molecules, suggests a large variety of of physiological functions beyond O2 storage/transport – in line with the fact that Hb are found in widely diverse living organisms such as plants, algae, animals, fungi, protozoans and bacteria - including anaerobic ones; indeed, in a number of these, biological roles as diverse as gas-sensors and enzymes have been assigned to Hb in addition to the classical O_2_-transporting role in vertebrates^1^.

Plant hemoglobins have recently been renamed as phytoglobins^2^. The first reported plant Hb were the leghemoglobins of soybean (*Glycine max*)^3,4^. These were found to be expressed in the nitrogen-fixing root nodule cell at high concentration (in the mM range) and bind O_2_, thereby playing a critical role in oxygen homeostasis with relevance to symbiosis with nitrogen-fixing bacteria^5^. Later, non-symbiotic plant hemoglobins (nsHbs) were discovered - but their function and mechanism of action are still unclear^6^. The nsHbs are divided into three classes, based on their primary structure, oxygen affinity and other structural aspects^3,5,6^. Class 1 nsHbs (including *Arabidopsis thaliana* (At) Hb1, AtHb1) exhibit a very high oxygen affinity and are expressed under stress conditions such as low temperature, hypoxia, or fungal infections^7,8^. Their overexpression is induced by hypoxia^5^ but is not directly influenced by a low oxygen concentration, hence indirectly by ATP levels^5,9^. The presence of class 1 nsHbs in growing tissues such as root tips of germinating seeds is correlated with the hypoxic stress as well^7^. Class 2 nsHbs (including AtHb2) have lower oxygen affinity^10–12^ and appear to play a role in young and developing plant tissues^13,14^. Apparently, the difference in oxygen affinity between class 1 and class 2 nsHbs is correlated to the Fe coordination sphere. Thus, while trans to the pervasive proximal histidine residue the vertebrate Hb feature no ligand at all in the ferrous state (Fe^2+^) and only an easily displaceable water/hydroxide in the ferric state (Fe^3+^), the ferric and as well as the deoxy ferrous AtHb1 and AtHb2 are hexacoordinated with a second amino acid^1,5,15,16^. The existence of a phenylalanine pressing on the distal histidine residue weakens the hexacoordination of AtHb1 - causing a “partial hexacoordination”^1,17,18^. Possible physiological roles for AtHb1 and AtHb2 as scavengers of reactive nitrogen species (RNS) including NO, or in other important metabolic pathways, have been proposed^19–21^. Class 3 nsHbs (including AtHb3) are known as “truncated hemoglobins” due to a shorter and compact ‘2-on-2’ structure which resembles bacterial globins^15^. This latter class of nsHbs is the least studied of all three and appears to be involved both in NO and peroxide-related pathways^22,23^.

In their oxy state, hemoglobins can undergo autoxidation, thus generating superoxide alongside the ferric form, methemoglobin - which can no longer bind O_2_^24–26^. Superoxide dismutase (SOD) then acts to remove the superoxo anion radical (O_2_^-•^), yielding hydrogen peroxide. The latter would normally be scavenged by catalases and peroxidases – but it can transform ferrous or ferric Hb into ferryl (Fe^4+^), generating toxic free radicals of physiological relevance^26^. The mechanisms that control the oxidation states of plant globins *in vivo*, though still unknown, may be deemed to be essential in understanding the physiological functions of these proteins, in light of the particularly facile redox and ligand exchange reactivity of their heme active sites. Physiologically-relevant antioxidants such as ascorbic acid, glutathione or NADH can be involved in such mechanisms^27–31^. Numerous factors such as oxygen pressure, pH, nucleophile concentrations, iron redox potential, protein conformation/mobility, and the presence of a distal histidine have been observed to affect the autoxidation process of hemoglobin and myoglobin^24,25,32^.

The present study presents data on three recombinant *Arabidopsis* phytoglobins in terms of their spectroscopic characteristics, redox state control, redox potentials and their relationship with autoxidation.

## Materials and Methods

### Cloning

The coding sequences of the three *Arabidopsis thaliana* nsHbs were cloned according to gene annotations at TAIR (www.arabidopsis.org) from cDNA similarly to procedures previously described^33,34^. The sequences were flanked by an N-terminal TEV recognition sequence (highlighted in italics), to allow cleavage of the affinity tag after purification, using the primers fwd_AtHb1 (5′-*GAGAATCTTTATTTTCAG*ATGGAGAGTGAAGGAAAGATTGTG-3′), fwd_AtHb2 (5′-*GAGAATCTTTATTTTCAG*ATGGGAGAGATTGGGTTTACAGAG-3′), fwd_AtHb3 (5′-*GAGAATCTTTATTTTCAG*ATGCAATCGCTGCAAGATAAGGC-3′), rvs_AtHb1 (5′-GGGGACCACTTTGTACAAGAAAGCTGGGTATTAGTTGGAAAGATTCATTTCAGC-3′), rvs_AtHb2 (5′-GGGGACCACTTTGTACAAGAAAGCTGGGTATTATGACTCTTCTTGTTTCATCTCGG-3′), and rvs_AtHb3 (5′-GGGGACCACTTTGTACAAGAAAGCTGGGTATTATTCTTCTGCTGGTTTATTGGCTGC-3′). A second PCR using the primer adapter (5′-GGGGACAAGTTTGTACAAAAAAGCAGGCTTAGAAAACCTGTATTTTCAGGGAATG-3′) was performed in order to amplify the construct for BP reaction and to clone into pDONR201 (Invitrogen) followed by an LR reaction by cloning into the vector pVP16^35^ (a kind gift of Russell L. Wrobel, Protein Structure Initiative (PSI) at the Center for Eukaryotic Structural Genomics (CESG), University of Wisconsin, Madison, USA). Recombination into this Gateway destination vector that contains an 8xHis:MBP (maltose binding protein) coding sequence of the Gateway cassette leads to an N-terminal 8xHis:MBP double affinity tag. The Hb genes (Hbx; x: 1–3) containing expression vectors pVP16::8xHis:MBP:Hbx were transformed in BL21 (DE3)) *Escherichia coli* cells by a heat shock procedure.

### Purification of recombinant proteins

A partial factorial design was employed for optimizing the expression of the nsHbs. In LB medium, the expression time (4, 8, 12, 24 h) along with the concentrations of IPTG (50, 100, 300, 500 µM) and heme (5, 15, 25, 50 µM) were varied while the temperature was kept constant at 30 °C. AtHb1 and AtHb2 were expressed as established by the optimization process while the AtHb3 expression protocol was partly modified. Thus, in the case of AtHb3 the cells were grown to a higher OD (1.2 compared to 0.4 for AtHb1 and AtHb2) and the temperature was reduced to 28 °C, after which 0.1 mM ferrous ammonium sulfate and 0.25 mM 5-aminolevulinic acid were added to the sealed culture vessel alongside IPTG together with 40 mL CO-purged LB medium. The cells were harvested by centrifugation at 4000 rpm for 20 min, resuspended in pH 7.8 buffer (50 mM sodium phosphate and 300 mM sodium chloride) and lysed by sonication followed by centrifugation at 15000 rpm for 30 minutes. The supernatant obtained after centrifugation was purified using a nickel affinity column (GE Healthcare Life Sci.) controlled by an ÄKTA purifier or using gravitational NiNTA resin columns. The proteins were eluted with 0.3 M sodium chloride, 50 mM Tris, 200 mM imidazole pH 7.8. While still bound to the column, carboxy-AtHb3 was oxidized to the ferric form using a 2 mM potassium ferricyanide solution. The eluates were loaded onto MBP Trap HP columns (GE Healthcare Life Sci.) and eluted with pH 7.8 buffer containing 10 mM maltose, 150 mM sodium chloride and 50 mM sodium phosphate. The final purification step was size-exclusion chromatography. For TEV cleavage and His8-MBP tag removal, the fusion protein was incubated overnight at 4 °C with a five-fold excess of TEV protease, expressed from pRK793^36^ (kind gift of David Waugh; plasmid ID: 8827, Addgene), in 50 mM sodium phosphate, pH 8.0, 0.5 mM EDTA and 1 mM DTT followed by HisTrap removal of the cleaved MBP, unreacted fusion protein and TEV protease. For further purification, a size exclusion and/or anion exchange step was also included. The protein fractions were analysed by 15% polyacrylamide SDS-PAGE. Analytical size exclusion chromatography was employed in order to assess possible oligomerization of the purified proteins.

### UV-vis and Raman spectroscopy

UV-vis spectra were recorded using a Varian/Agilent Cary 50 UV-Vis spectrophotometer. The molar extinction coefficients of the Soret, α and β bands of the studied globins were determined using the alkaline hematin method for quantifying the heme^37^. For this purpose, a reagent which consists of nonionic surfactant (5% Triton X-100) dissolved in 0.2 M sodium hydroxide was used. Equine heart myoglobin (Sigma-Aldrich) in a 2–12 μM concentration range was employed for the calibration curve. Resonant Raman spectra were recorded using a Renishaw inVia Raman Microscope at 50% of the 80 mW HeCd laser, emitting at 442 nm. The Raman back-scattered light was directed to a spectrometer equipped with 1800 lines/mm grating and a CCD detector with a spectral resolution of ~4 cm^−1^. The instrument was calibrated prior to each experiment using a silicon sample as an internal standard. The spectra were recorded with exposure times of 5–10 seconds and two accumulations. The protein concentration was adjusted to 100 µM and the buffer was sodium phosphate pH 7.4. The experiments were done in triplicates. Met forms were obtained by potassium ferrycyanide treatment of the as-purified forms followed by PD-10 column desalting. The carboxy form was prepared by a procedure described elsewhere^38^. DeoxyHb was freshly prepared from the as-purified form using a slight excess of dithionite followed by desalting on a PD-10 column, and was kept on ice in order to minimize autoxidation.

### Redox potential determination

Redox titrations for 8 μM nsHb (AtHb1, AtHb2, AtHb3) and bovine Hb in the range of pH 5–10 were performed in 3-mL anaerobic quartz cuvettes at 25 ± 2 °C under an argon atmosphere. A 100 mM three-component buffer (phosphate, boric acid and sodium acetate) was employed. The buffer was deoxygenated prior to the measurements, by bubbling with argon. The titrations were performed in the reductive direction, from the ferric to the ferrous form, with a freshly prepared 5 mM dithionite solution and using an additional volume of 1 μL per step. To facilitate electrical communication between the protein and electrode, six mediators were added: hexaammino ruthenium (III) chloride (−221 mV), naphthoquinone (−102 mV), 2,6-dichlorophenolindophenol (−10 mV), phenoxazine (33 mV), potassium ferricyanide (213 mV), and ferrocenecarboxylic acid (287 mV) - with final concentrations of 1 μM each; these mediators were previously checked for their stability and reversibility. For the UV-vis spectral monitoring, a modular spectrometer (model UV-VIS USB4000), a miniature UV-VIS-NIR light source (model DT-MINI-2-GS), 2 UV optical fibres (model QP600–025-SR), a quartz cuvette of 1 cm lightpath length (model CV-Q-10) and a cuvette holder (model CUV-UV), all from Ocean Optics (USA), were used. The Pt working electrode (diameter of 0.5 mm, length 1 cm), the reference electrode (Ag/AgCl/KCl 1 M), the purge tube (ethylene tetrafluoroethylene made, outer diameter of 1.6 and inner diameter of 0.5 mm) and the injection tube (polyimide coated quartz capillary, outer diameter 0.5 mm, inner diameter 0.05 mm and length of 50 cm) were fixed in a SEC-C Teflon cap (model EF-1359, BASi, USA). For the injections of standard solutions, a computer-controlled peristaltic pump (model Reglo Digital MS-2/8, Ismatec, Switzerland) equipped with a 3-stop Tygon LFL tube (inner diameter of 0.38 mm) was used. During the additions and measurements, the solution inside the cell was continuously stirred using a miniature polytetrafluoroethylene-encapsulated magnetic stirring bar and a magnetic stirrer (model FB15001, Fisher Scientific). The electrochemical measurements were completed using a home-made P/G-Stat/electronic voltmeter controlled by computer via a PCI-6259M data acquisition board (National Instruments, USA). The control of all equipment and the synchronous acquisition of spectrophotometric and electrochemical data were done using dedicated LabView 2015 (National Instruments, USA) software applications.

### Autoxidation and reduction assays

The pH influence upon autoxidation was evaluated using a 0.1 M four-component buffer containing boric acid, sodium citrate, TAPS and sodium phosphate. Autoxidation was further monitored at various concentrations (10, 50, 100, 150, 300, 400, 500 and 600 mM) of potassium sulfocyanide, sodium chloride, sodium fluoride and potassium bromide as well as in the presence of 1.25, 5 and 8.75 mM ascorbate, glutathione and NADH (Sigma Aldrich, Germany), in 50 mM sodium phosphate buffer, pH 7. Half-life (t_1/2_) was used as a measure of the autoxidation tendency and was calculated based on the kinetic constant obtained by fitting the data to exponential decay first-order functions on the UV-vis spectral data. The superoxide anion released during autoxidation was detected using a cytochrome *c* reduction protocol^39,40^. Thus, 30 µM cytochrome *c* were mixed with 25 µM oxyHb, 50 µM catalase (CAT) and with or without superoxide dismutase (SOD). The reduction of cytochrome *c* was monitored at 550 nm. Hb reduction reactions were carried out in the presence of ascorbate, glutathione and NADH at several concentrations in pH 7, 50 mM sodium phosphate. ANOVA and t-test were applied for statistical significance testing using Statistica 8 software (*SatSoft* USA).

## Results and Discussion

### Expression, purification and spectroscopic characterization

A partial factorial experimental design was employed for recombinant overexpression of all three non-symbiotic hemoglobins from *Arabidopsis thaliana*. The optimum concentrations of IPTG and heme as well as the proper expression duration (all dependent on amino acid sequence, size, hydrophobicity, isoelectric points, etc.)^41^ were examined for the three *Arabidopsis* Hbs. Typical for cases where the rate of the protein synthesis is lower than the folding rate (especially with the problem of cofactor insertion), the temperature was kept constant at 30 °C or below. For Hb as well as for other proteins, this prevents the formation of aggregates or inclusion bodies^41^. Addition of heme to the culture medium helps hemoglobin achieve proper folding – since *E*. *coli* may otherwise fail to synthesize heme in the amount and at rates compatible with the synthesis of the globin polypeptide chains. In order to prevent its degradation, heme was added in two stages. The hemoglobin yield was increased by 12% when proper amounts of heme and IPTG were added to the culture medium. The optimum conditions were established using a simplified factorial experimental design: 110 μM IPTG and 60 μM heme for AtHb1, 150 μM IPTG and 40 μM heme for AtHb2, 200 μM IPTG and 30 μM heme for AtH3. (Fig S1).

**Figure 1:**
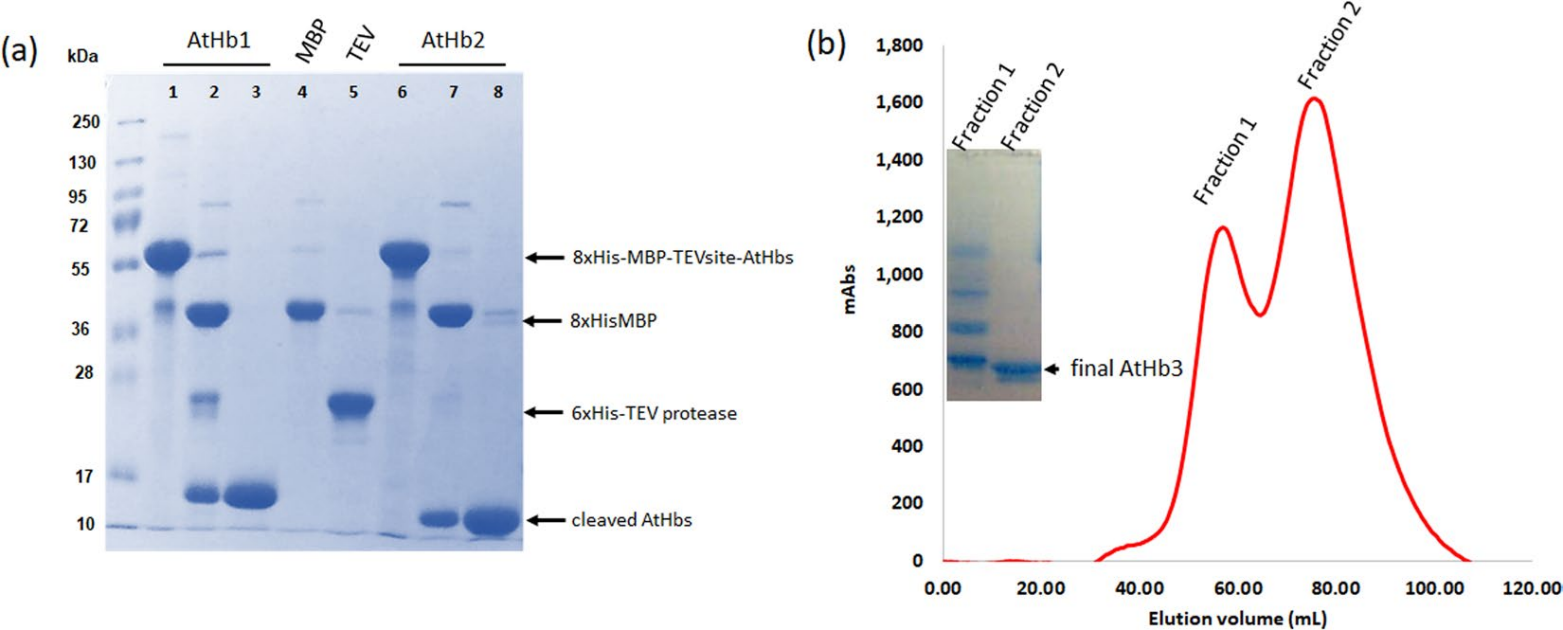
Preparation of pure recombinant phytoglobins. SDS-PAGE showing the TEV mediated His8-MBP removal for AtHb1 and AtHb2 (**a**) and final anion exchange purification of AtHb3 (**b**). ‘MBP’ stands for cleaved recombinant His8-MBP affinity tag and ‘TEV’ for recombinant His6-TEV protease. Corresponding full-length gels are shown in Figure S4.

His8-MBP-tagged hemoglobins yielded higher amounts of soluble protein compared to the simple His-tagged versions (data not shown). Preliminary experiments showed that the His8-MBP tag is removed easily from His8-MBP:AtHb1, reasonably well in the case of His8-MBP:AtHb2 but poorly in the case of His8-MBP:AtHb3 (Fig S2). The latter protein also presented dimers or oligomers, after HisTrap purification and before cleavage. This was much improved after CO was used during expression, as suggested by others^15^; without CO, the quality of His8-MBP:AtHb3 before cleavage was very poor. This could be an indication of ROS generation (in cell or during purification steps) that leads to oxidative protein coupling - which would indeed be diminished by CO due to its high affinity for the ferrous heme iron which would place to protein in the ferrous-carboxy form and hence limit the autoxidation or the ensuing ROS generation. After completing the chromatographic procedures for purification, SDS-PAGE electrophoresis showed a higher purity degree for AtHb1 and AtHb2 (Fig. 1a). However, in the case of AtHb3 the cleaved protein still presented oligomers that were easily removed using anion exchange chromatography (Fig. 1b). The purified proteins were further checked for their apparent native size and possible oligomerization in solution, using analytical size exclusion chromatography (Fig. S3). Both AtHb1 and AtHb3 appear as dimers before as well as after His8-MBP removal, while AtHb2 is a monomer both before and after His8-MBP cleavage. These findings are in good agreement with already known data^42–44^ and indicate that the His8-MBP tag does not hinder intermolecular interaction or structurally affect the globins.

**Figure 2:**
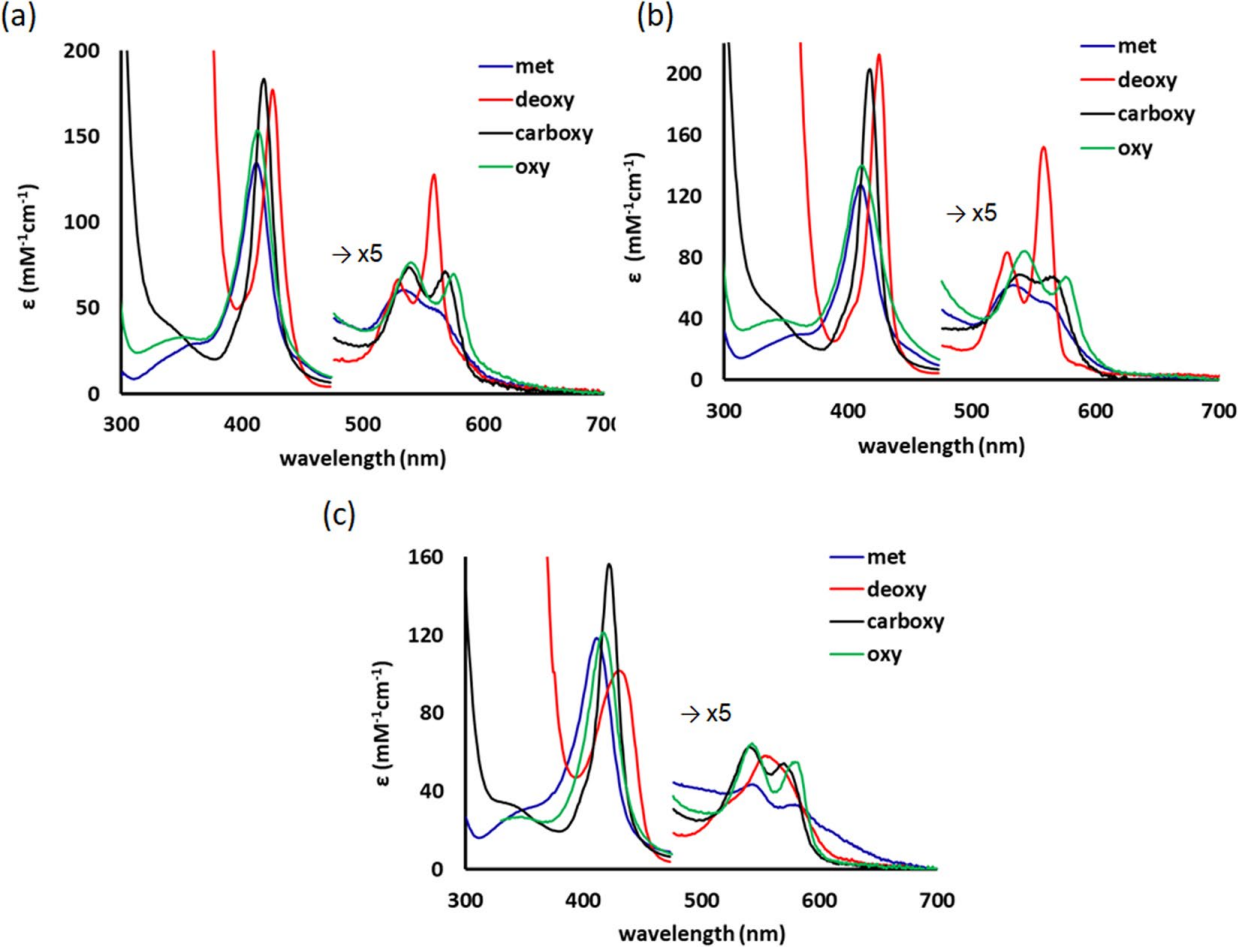
Optical spectra of pure recombinant phytoglobins - met, deoxy, carboxy and oxy AtHb1 (**a**), AtHb2 (**b**) and AtHb3 (**c**) in phosphate buffer, pH 7.4.

Following their purification, the procedure for heme determination of the three nsHbs was performed using the alkaline method (Fig. S5); their corresponding extinction coefficients are presented in Table 1. Good agreement was found between already known data as well as for bovine hemoglobin^15^. Figure 2 presents optical absorption spectra of met, carboxy, deoxy and oxy forms for the three nsHbs.

**Table 1:** Extinction coefficients of pure recombinant phytoglobins in the oxy, met and deoxy forms (in phosphate buffer, pH 7.4).

Hb	AtHb1			AtHb2			AtHb3			bovine Hb		
	ε (mM^−1^cm^−1^)			ε (mM^−1^cm^−1^)			ε (mM^−1^cm^−1^)			ε (mM^−1^cm^−1^)		
form	oxy	met	deoxy	oxy	met	deoxy	oxy	met	deoxy	oxy	met	deoxy
Soret	150	133	175	140	127	212	120	116	98	128	142	134
	(*415*)	(*413*)	(*426*)	(*412*)	(*411*)	(*425*)	(*418*)	(*413*)	(*433*)	(*414*)	(*406*)	(*430*)
α	15	12	13	17	12	16	13	9	12	14.6	9	13
	(*540*)	(*535*)	(*530*)	(*544*)	(*535*)	(*530*)	(*544*)	(*545*)	(*555*)	(*542*)	*(*542)	(*558*)
β	14	9	26	13	10	30	11	7	—	14.8	7	—
	(*576*)	(*565*)	(*560*)	(*578*)	(*565*)	(*559*)	(*582*)	(*584*)		(*577*)	(*578*)	

In parentheses, the spectral maxima in nm are also indicated for each band.

The spectra of the deoxy form of the AtHb1 and 2 present characteristics typical for hexacoordinated hemoglobins with a bishistidine iron coordination: a well-defined high-extinction coefficient Soret band and distinctly split α and β bands. AtHb2 exhibits a stronger hexacoordination as judged from the higher absorptivities of spectral bands and higher β/α intensity ratio^44^. These two globins are also hexacoordinated in the met form, based on spectral position of the Soret band (413 nm) and the spectral profiles of the α and β bands that indicate a low-spin iron, typical for bishistidine coordination^44,45^. By contrast, the AtHb3 spectra are consistent with a pentacoordinated hemoglobin – i.e., a single band at ~550 nm in the deoxy form and high-spin iron fingerprints, that are more enhanced at acidic pH when the aqua met form predominates (band > 620 nm) since the pKa of the water-iron bound molecule is 7.17 for AtHb3^15^. Minor visible differences between the three globins for the carboxy and oxy forms are also noted, especially in the α and β bands region.

**Figure 3:**
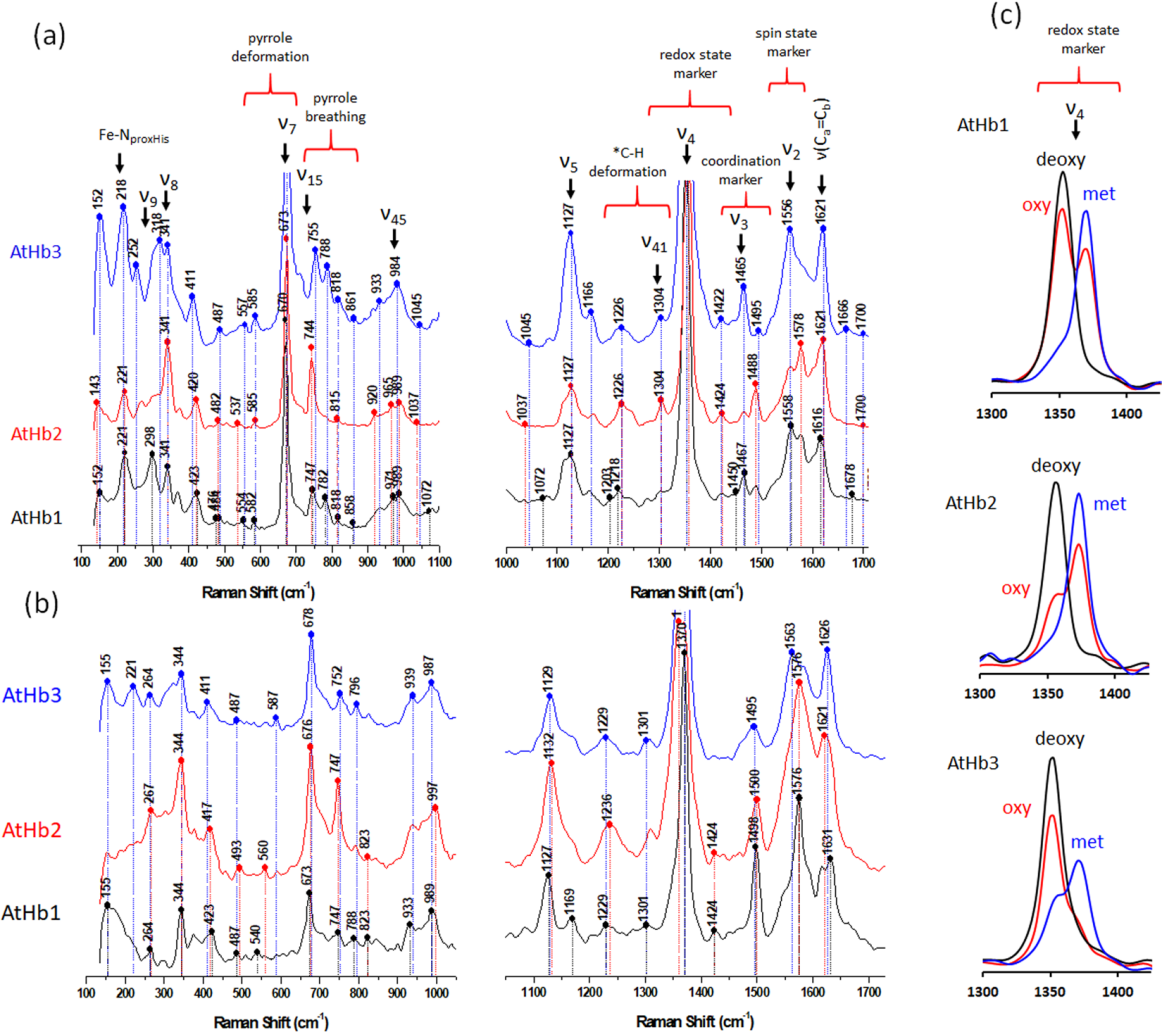
Resonance Raman spectra of pure recombinant phytoglobins. Resonance Raman spectra of the three studied nsHbs, in the deoxy **(a)** and met **(b)** forms - low-frequency (left) and high-frequency (right) regions. The highest intensity vibrational mode (ν_4_) for ferrous, ferric and oxy forms of all three globins (**c**). The laser-excitation wavelength was 442 nm, and the proteins were solved in phosphate buffer, pH 7.4.

Resonance Raman (RR) spectra of the three studied nsHbs are presented in Fig. 3 and as well as, in all three forms, in Fig. S6. The observed vibrational bands were assigned according to already available data^43,46,47^. The low-frequency region of the RR spectra (100–1000 cm^−1^) shows several in-plane and out-of-plane vibrational modes of the heme cofactor and possible ligand vibrational modes. The 218 cm^−1^ band is assigned to the Fe-N(proximal His) stretch; this is strong in the case of deoxyAtHb3 and is replaced by a 221 cm^−1^ band in the case of AtHb1 and 2. This band is stronger for pentacoordinated hemoglobins but can also appear in the hexacoordinated hemoglobins or other hemeproteins at wavenumbers up to 240 cm^−1^, depending on the charge of the proximal imidazole^43,48^. A lower value would indicate a less charged proximal imidazole or a stronger dislocation of the iron from the heme plane, therefore explaining their low activity in the met form. Noticeable dissimilarities may be observed in the 295–425 cm^−1^ region, the ν_9_ mode (~300 cm^−1^) and the ν_8_ mode (340–345 cm^−1^), indicating the existence of important conformational differences that cause major out-of-plane distortions in the heme and could have significant implications for their reactivity. Regarding the 550–850 cm^−1^ region where pyrrole deformations and pyrrole breathing modes are observed and hemoglobins usually present high similarities, AtHb2 appears as totally different than the other two counterparts suggesting a totally different structure with distinct activity implications as shown before^6,49,50^ and presented in this study as well. Another important region of the RR spectra of heme proteins is the high-frequency region (900–1.700 cm^−1^) that contains porphyrin in-plane vibrational modes, which are well-known indicators of the oxidation state (ν_4_), geometry or coordination state (ν_3_), and the spin state (ν_2_) of the heme iron^43,47,51^. All ferric hemoglobins display a frequency of the electron density marker specific for oxidation state (ν_4_), 1370 cm^−1^, a frequency characteristic of the met form and for the deoxy forms at 1353 cm^−1^ which is specific for ferrous state. Interestingly, the spectra of the oxy forms exhibit a mixture of states, a good marker for the strength of the iron-dioxygen bond strength and implicitly of the superoxide-ferric character (Fig. 3c). The ratios of the two bands are well associated with autoxidation rates via superoxide displacement (*vide infra*). In the case of the ferrous forms, very active bands at ~1465 cm^−1^ and ~1555 cm^−1^ for AtHb3 clearly show a dominant high-spin pentacoordinated state in contrast with the bands of AtHb2 at ~1489 cm^−1^ and 1580 cm^−1^, indicating a strong low-spin hexacoordinated state - while AtHb1 exhibits both of these two bands and is hence interpreted to feature a mixture of two types of molecule populations, the hexacoordinated form dominating over the pentacoordinated form. All these are in good agreement with other published data for the same or similar hemoglobins^6,50,52^. In the case of the ferric forms, AtHb3 has an increased low-spin hexacoordinated character compared to the ferrous form due to the hydroxo met formation at this pH (7.4) while the other two globins show similar spin and coordination states as expected and previously shown^53^.

### Redox potential determination

**Figure 4:**
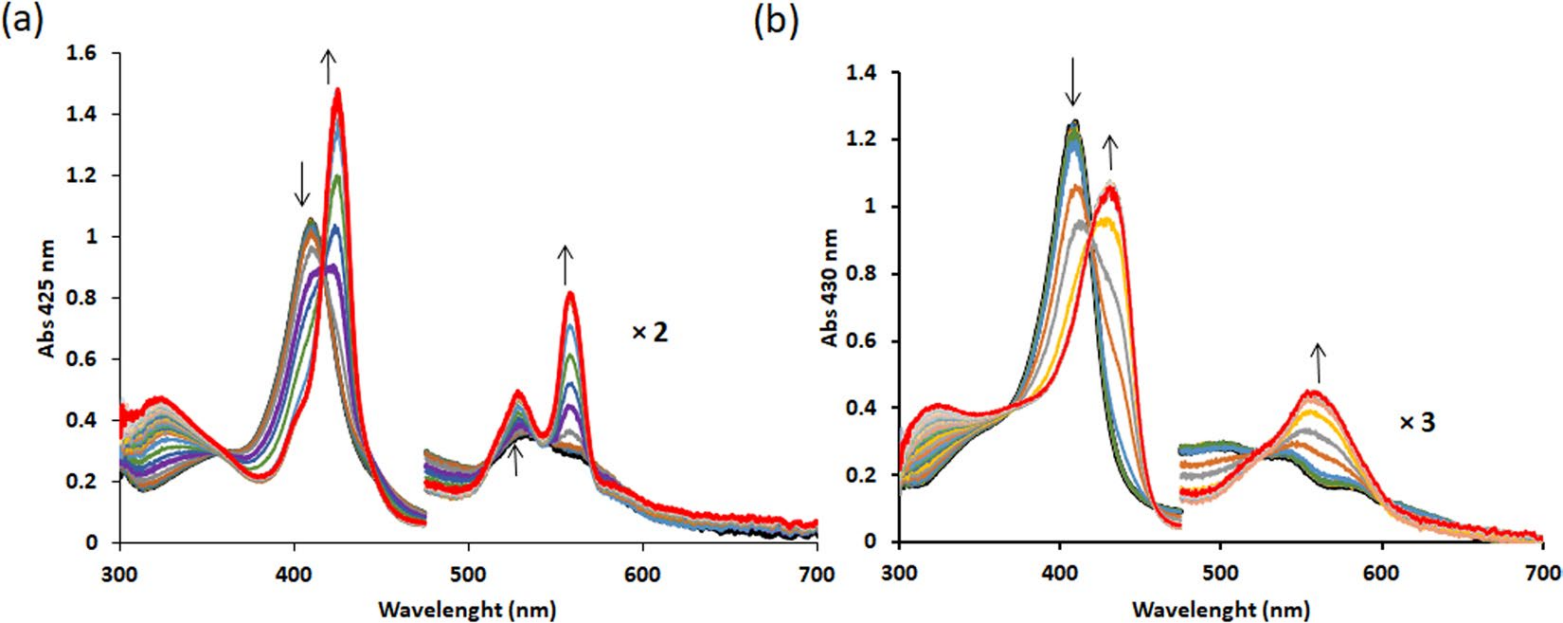
Spectroelectrochimistry redox titration of pure recombinant phytoglobins. Spectroelectrochemistry redox titration of AtHb2 (**a**) and AtHb3 (**b**) at pH 7 with dithionite. Arrows indicate the directions of absorption change at different wavelengths occurring in reductive direction at 25 ± 2 °C.

**Figure 5:**
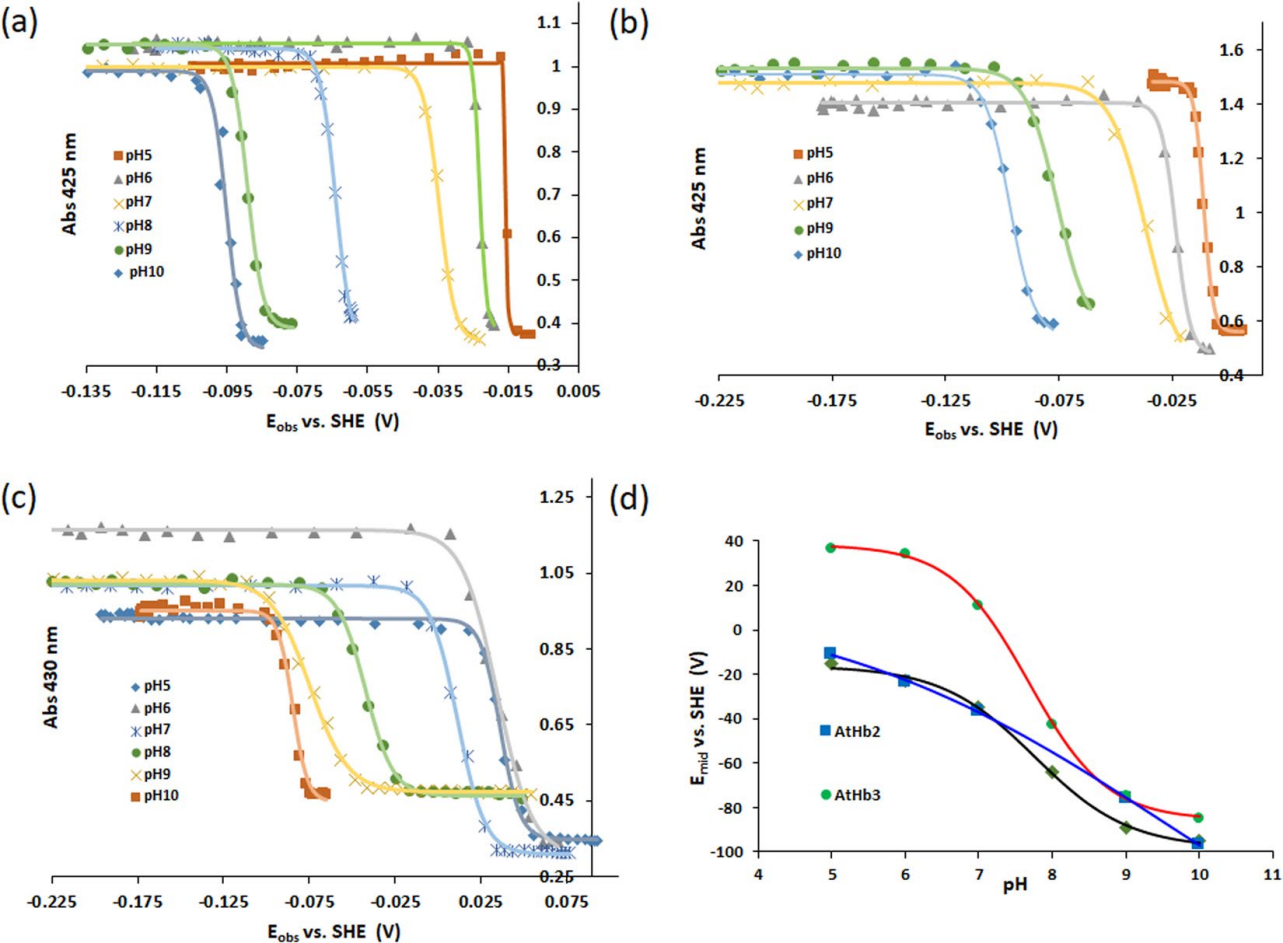
Redox potential determination of the pure recombinant phytoglobins. Dependence of the absorbance for AtHb1 at 425 nm (**a**), AtHb2 at 425 nm (**b**) and AtHb3 at 430 nm (**c**) vs. reduction potential. The monitored absorbance corresponds to the ferrous form of the hemoglobin. Dependence of mid redox potential *versus* pH for AtHb1 AtHb2 and AtHb3 (**d**).

The heme iron in globins can generally be cycled through ferrous (Hb-Fe^2+^), ferric (Hb-Fe^3+^) or ferryl (Hb-Fe^4+^) oxidation states, with the less usual super-reduced iron and sulfo-ferryl forms proposed in special cases^54,55^. The midpoint redox potential for the Fe^3+^/Fe^2+^ transition of the heme of AtHb1, AtHb2 and AtHb3 was determined by spectrophotometric titration with dithionite in the 5–10 pH range. The typical absorption changes during the titration and transformation of the ferric Hb-Fe^3+^ to the ferrous forms of AtHb2 and AtHb3 at pH 7 are shown in Fig. 4. As expected, well-defined isosbestic points are observed. Dependencies of the absorbance at the Soret band versus the observed redox potential at different pH values (pH 5–10) are shown in Fig. 5a–c and the calculated midpoint redox potentials are presented in Fig. 5d.

AtHb1 and AtHb2 exhibit similar redox potentials, at all pH values, with the highest values in the acidic range (−15 mV for AtHb1 and −11 mV for AtHb2) and the lowest values in the alkaline range (−95 mV for AtHb1 and −97 mV for AtHb2). In the case of AtHb3, the midpoint redox potential has more positive values than for AtHb1 and 2; however, in the alkaline region the potentials become comparable with the ones of AtHb1 and 2 where the heme is low-spin and hexacoordinated (hydroxy-met). Moreover, AtHb1 exhibits a slight sigmoidal dependence upon pH, similarly to AtHb3, slowly leveling off towards very acidic or alkaline values – while the AtHb2 dependence upon pH is rather linear (Fig. 5d), most probably due to its partly pentacoordinate character. Although our values are somewhat more positive than expected, all these are sound indications of the more negative redox potentials for low spin, hexacoordinated states as was previously shown in the case of Ngb, Cgb, rice Hb1 and bishistidine H64V/V68H Mb^56,57^. Therefore, the different coordination strength, both in the ferric and ferrous oxidation states, leads to different reduction potentials in globins – the hexacoordinated forms having more negative potentials due to tighter binding in the ferric oxidation state.

Besides the coordination strength, it was shown that critical roles in redox potential as well as autoxidation (*vide infra*) are played by key residues in the distal binding pocket at the heme. Firstly, a more tightly bound E7 residue (His in the hexacoordinated AtHb1 and 2 and in mammalian hemoglobins) in the ferric versus ferrous state explains a lower redox potential. An important role may be played the the B10 residue, which is a conserved Phe in hexacoordinated hemoglobins AtHb1 and 2 and is known to affect both hexacoordination and autoxidation^58,59^. A mutation of Phe-B10 augments the hexacoordination in the ferric state relative to the ferrous state, thus lowering the redox potential^58^. By contrast, the different behavior of AtHb3 regarding its lower redox potential, especially at acidic pH, could also be explained by different residues in the binging pocket. Here, Phe-B10 is replaced by a Tyr and His-E7 by a Gln. The Tyr-B10/Gln-E7 combination in AtHb3 is known to be involved in exogenous ligand stabilization^60^ and is similar to those of flavoHbs that are efficient for the NO dioxygenase activity. In contrast to hexacoordinated hemoglobin, these residues might be involved in controlling the hexacoordinated alkaline transition that takes place in the ferric form of AtHb3, similarly to what is seen in cytoglobin which in addition presents an important Arg in the E10 position and resembles more to pentacoordinated hemoglobins rather than hexacoordinated both structurally and based on redox potential value^61,62^. Redox titration data indicated that in cytoglobin the iron ligands are switched depending on the redox oxidation state of the iron^62^ and, moreover, its hexacoordination was shown to be influenced by lipid binding and thus indicating a role in lipid signaling of oxidative stress^63^. Similarly, though in totally different conditions, an induced transitient hexacoordinated state in the ferrous form of AtHb3 was also observed^11^.

### Autoxidation – pH dependence and superoxide implications

**Figure 6:**
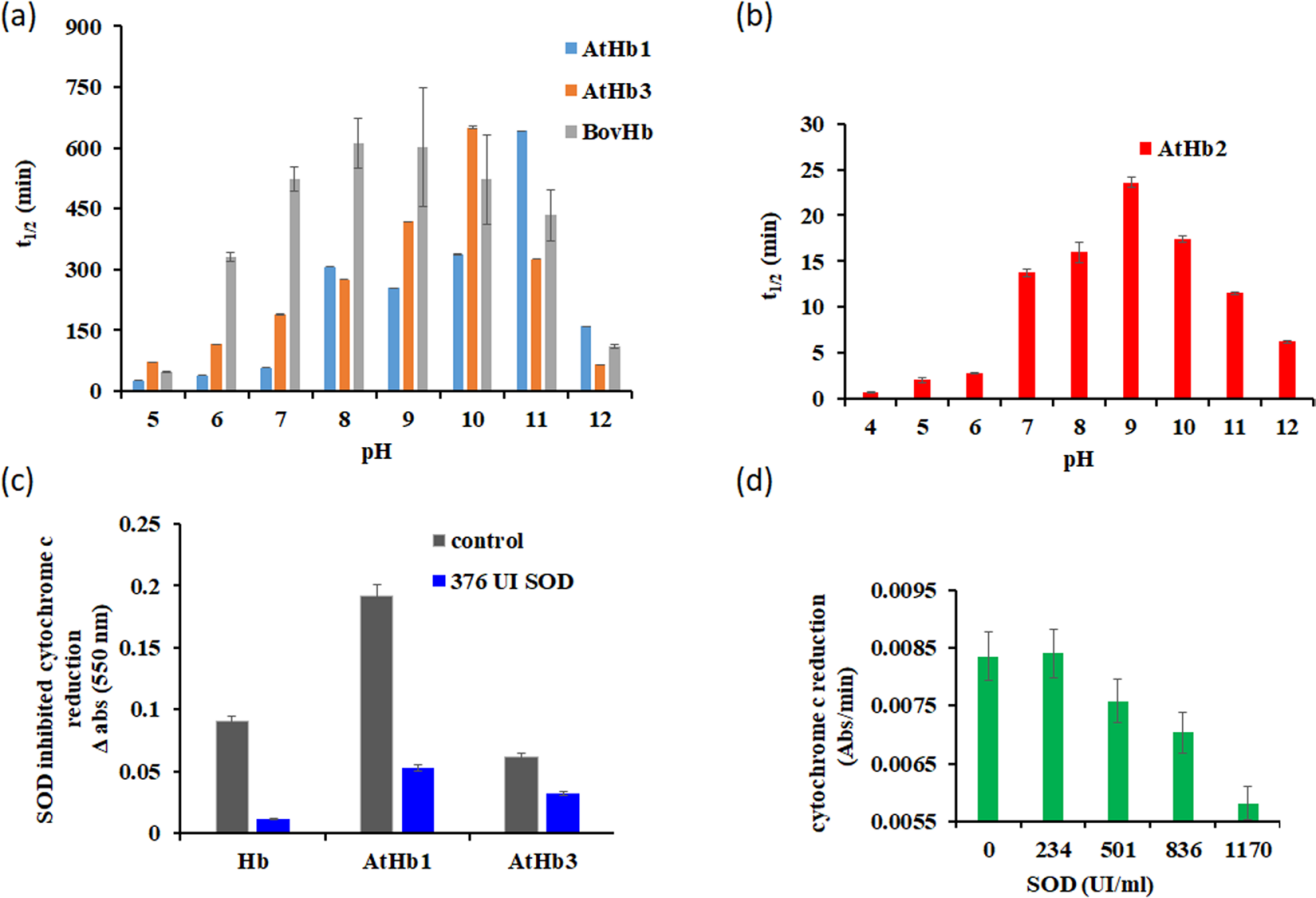
Autoxidation of phytoglobins dependent upon pH. Autoxidation (t_1/2_) dependence upon pH was measured for AtHb1, AtHb3, bovHb (p < 0.001, ANOVA) (**a**) and AtHb2 (p < 0.001) (**b**) in phosphate buffer at room temperature. SOD inhibits cytochrome *c* reduction in the presence of oxyAtHb1, AtHb3 and bovine Hb, as reflected in the difference of absorbance at 550 nm before and after reaction is complete (p < 0.001) (**c**) and in the initial rate of cytochrome *c* reduction for AtHb2 (p < 0.01) (**d)**.

In order for phytoglobins to act as NO scavengers via an NO dioxygenase reaction that leads to nitrate, they have to be able to bind molecular oxygen^64,65^ – since the ferrous-dioxygen adduct is responsible for the direct reaction with NO in NO dioxygenases^66^. It generally accepted that the Hb-dioxygen complex is best described as HbFe^3+^-O_2_^-•^ – although upon dissociation of dioxygen the heme iron remains in the ferrous state and will rarely dissociate to superoxide (O_2_^−•^) and Fe^3+^ heme (autoxidation). The resulting ferric (met) form does not have the ability to bind molecular oxygen and is hence inactive for O_2_ binding and implicitly also for NO dioxygenation^67^. High autoxidation tendency is generally correlated with a more solvent-accessible site (likely also richer in polar aminoacids) as well as with a weaker hydrogen bond between the distal histidine and the oxygen molecule^17^. Half-life times of the autoxidation of the three *Arabidopsis* hemoglobins and of bovine hemoglobin are shown in Fig. 6a,b. These show an expected pH dependence in both acidic and alkaline ranges, substantially different from protein to protein and arguably related to the hydrophobicity of the heme pocket. All three hemoglobins show a parabolic profile dependence on pH. At a low pH, the distal histidine becomes protonated and isno longer able to efficiently stabilize the dioxygen ligand through a hydrogen bond. This leads to a rapid loss of superoxide and hence higher autoxidation rates. At a higher pH, the protein tertiary structure changes also appear to promote autoxidation. In the case of human hemoglobin, kinetic and thermodynamic studies have revealed that autoxidation is due to nucleophilic substitution of oxygen by a water molecule or hydroxide ion^32^. Of the three *Arabidopsis* nsHb, AtHb2 shows the greatest autoxidation tendency, with a half-life time of 23.6 minutes at pH 9. The crystal structures of AtHb2 and AtHb1 reveal hexacoordinated states, with the average distance between the iron atom and distal histidine at 3.04 Å for AtHb2 and at 2.90 Å for AtHb1^68^. It may be argued that the strength of the hydrogen bond formed between the distal histidine and oxygen would not play a meaningful role in autoxidation. On the other hand, the longer Fe-(distal His) distance in AtHb2 may be an indicator of a wider and more solvent-accessible active site – which would fit with the autoxidation trends. The Phe-B10 residue in the vicinity of His-E7 was shown to be involved in controlling autoxidation by protecting against solvent access; thus, hydroxide anion access in the heme pocket is two orders of magnitude higher for a PheB10Leu mutant compared to the wild-type^59,69^. It was observed that autoxidation rates are also dependent upon the coordination state of the hemoglobin iron atom, increasing as the iron coordination number (not counting solvent molecules) increases^70^; as also, the electron transfer rate is higher in hexacoordinated hemoglobins^71^. In our data, AtHb2 shows the largest autoxidation tendency, followed by AtHb1 which is only partially hexacoordinated and followed by AtHb3 and bovine hemoglobin which are both pentacoordinated. The strength of the distal histidine – iron atom bond being stronger for the hexacoordinated globin, superoxide vacates its heme pocket more easily. The redox potential could be important here to a lesser extent, since AtHb1 and 2 have comparable redox potentials but much different autoxidation rates. Thus, autoxidation of the hexacoordinated hemoglobin AtHb2 is favored compared to the less hexacoordinated (AtHb1) or the pentacoordinated ones (AtHb3 and BovHb); this also implies a significant role for the distal histidine in the autoxidation reaction. Interestingly, in the RR spectra, the strength of the shift in the vibrational mode specific for oxidation state (ν_4_) from 1353 cm^−1^ (deoxy) to 1370 cm^−1^ (met) is well correlated with the autoxidation rate; it is possible that here the laser light is responsible for accelerating the superoxide dissociation (Fig. 3c). However, despite the availability of a large amount of, the mechanism of autoxidation is not completely understood^44,72,73^.

Superoxide and its product peroxide generated by spontaneous dismutation are mainly responsible for the oxidation of the iron during autooxidation. It was shown that one third of the rate of conversion of oxyHb to metHb is due to peroxide^74^. Superoxide reacts with oxyhemoglobin and methemoglobin at approximately the same rate and therefore it cannot determine complete reduction or oxidation^75^. The generation of superoxide during autoxidation of the three *Arabidopsis* hemoglobins is shown in comparison to a mammalian Hb in Fig. 6c,d, as measured by a cytochrome *c* reduction experiment^24,39,40^. By incubating cytochrome *c* and oxy-hemoglobin, cytochrome *c* is reduced at a rate correlated with the rate of superoxide generation and therefore autoxidation of hemoglobin. The inhibition of cytochrome *c* reduction by the superoxide dismutase (SOD), shown in Fig. 6c,d, indicates that superoxide is responsible for the cytochrome *c* reduction and therefore superoxide is directly involved in controlling the autoxidation rates, beyond being a mere product of the reaction. OxyAtHb2 causes the fastest cytochrome *c* reduction reaction, well correlated with its autoxidation rate; also here, the initial reduction rate is slower as the SOD concentrations are increased (Fig. 6d). An insufficient amount of catalase added in the AtHb2 experiment causes reoxidation of cytochrome *c* at a rate dependent of the concentration of the administrated SOD, as a further proof for superoxide production and dismutation to peroxide during the autoxidation process (Fig. S7A).

The kinetic profile of cytochrome *c* reduction in the case of the other three hemoglobins (AtHb1, AtHb3 and bovHb), which present a lower autoxidation rate, is different. The reduction of cytochrome *c* in the presence of AtHb1 follows the same kinetic pattern as bovine oxyhemoglobin and generates the highest amount of reduced cytochrome *c*. Without SOD, the reaction starts with a rapid increase in absorbance at 550 nm, specific for cytochrome reduction, follow by a lag phase and ending with another phase characterized by an increase in absorbance. In the presence of SOD there was only a little amount of reduced cytochrome generated (Fig S7). In the case of AtHb3, the kinetic profile is mostly different, but the inhibition of the reduction of cytochrome *c* is clearly observed.

The main important physiological role of phytoglobins is best known for class 1 and implies cellular NO scavenging since they are rapidly induced in hypoxia, increasing plant survival in such stress conditions for several species such as maize, *Arabidopsis* and alfalfa^7,20,76,77^. In order to keep NO metabolism functional for a physiologically relevant time, hemoglobins have to be resistant to autoxidation or need an efficient electron donating partner to reduce back the ferric hemoglobin generated during autoxidation; the latter option would be an energy consuming situation. The fact that AtHb2 presents such a high autoxidation rate and so distinctly high superoxide generation rate compared to AtHb1 and AtHb3 (Figs 6, S7) indicates its implication in a totally different biological role, besides that of NO scavenging. Indeed, other studies have shown that, in contrast to AtHb1, AtHb2 is induced by low temperature^10^ or cytokinins^78^ and is preferentially expressed in developing organs^79^, increases oil accumulation in *Arabidopsis* seeds^13^ and regulates the synthesis and transport of auxins by altering the level of NO in specific cells^80^. In addition, in light of our findings, the previously hypothesized roles of AtHb2 as molecular oxygen transporter is also less probable^68^.

### Autoxidation in the presence of anions

Figure S8 shows the autoxidation t_1/2_ for all four hemoglobins in the presence of thiocyanate, chloride and fluoride at various concentrations. Each of these anions alters the autoxidation rates differently. For mammalian myoglobin and hemoglobin, anion-induced autoxidation proceeds through a nucleophilic attack by the anion, and therefore the autoxidation rates increase with anion nucleophilicity: SCN^−^ > F^−^ > Cl^−^^24,25,32^. A clear dependence of autoxidation rates upon all anion concentrations was observed in the case of AtHb2. Here, both thiocyanate and fluoride accelerate the autoxidation even at lower concentrations (10 mM each). At higher concentrations, thiocyanate has a significant influence on the autoxidation, more than the other two anions. Hence, it can be concluded that AtHb2 autoxidation in the presence of anions follows the order SCN^−^ > F^−^ > Cl^−^ and proceeds through a bimolecular substitution mechanism, similarly to bovine hemoglobin^32^. AtHb1 and AtHb3 are more resistant to autoxidation in the absence as well in the presence of anions. In their cases, only thiocyanate promotes autoxidation - more so in the case of AtHb1. For AtHb3 this effect was observed only above 300 mM. Contrary to what was expected, fluoride and chloride stabilize AtHb1 and AtHb3 respectively, towards autoxidation.

It was shown that class 1 phytoglobins are involved in high salinity response^81^ and their function as NO scavengers is not affected by high salt concentrations^82^, in line with our findings which show that the AtHb1 autoxidation rate and therefore its NO dioxygenase activity is not affected by the high halide/salt concentration. No data exist regarding class 2 phytoglobins’ involvement in high salinity stress, to our knowledge. However, since AtHb2 autoxidation rates are strongly influenced by even small increases of salinity, our data suggest that AtHb2 activity could be affected by high salt stress conditions.

### Mitigating autoxidation by electron donors

**Figure 7:**
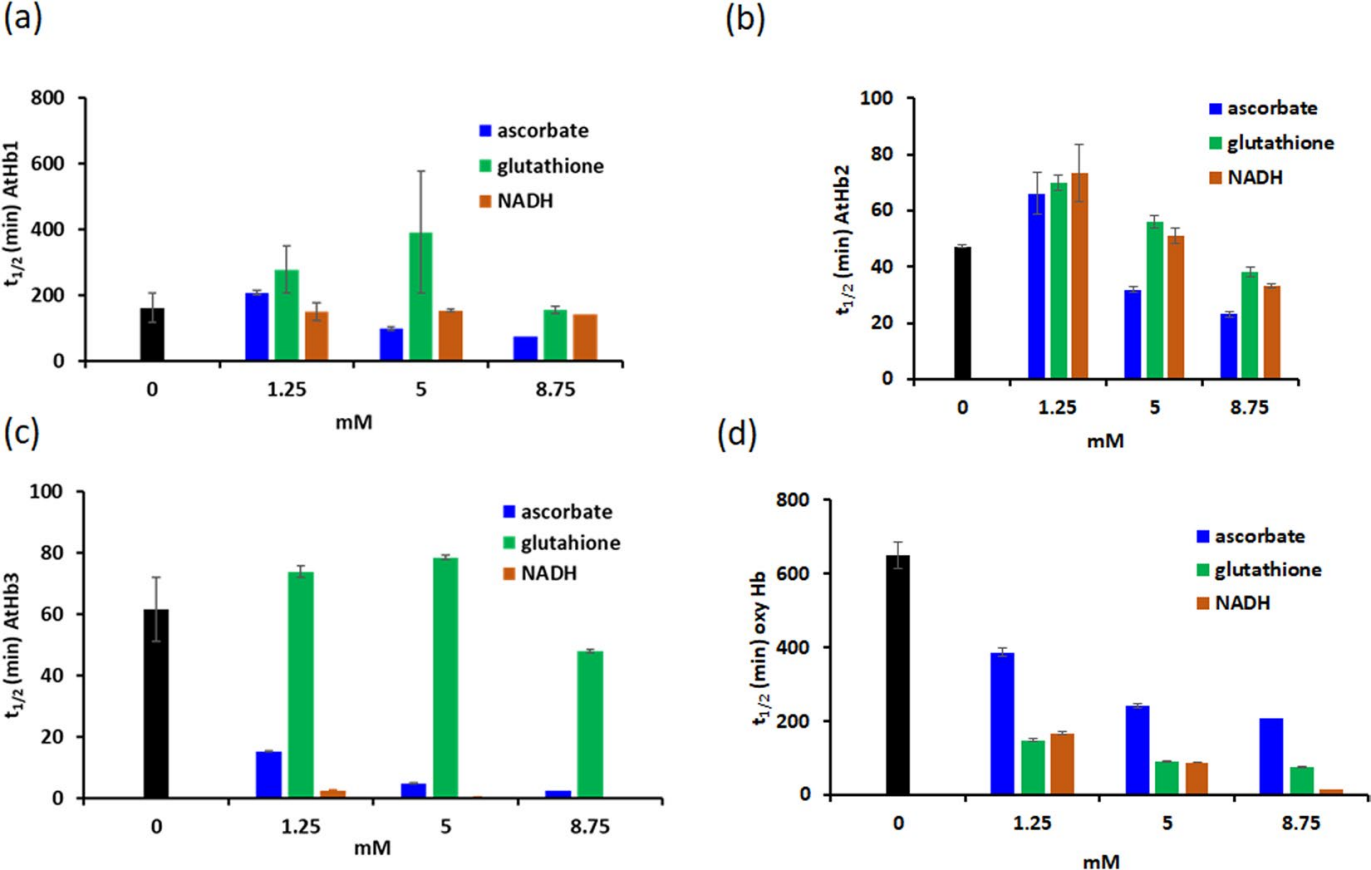
Autoxidation of phytoglobins dependent upon reducing agents. Autoxidation (t_1/2_) in the presence of ascorbate, glutathione and NADH for AtHb1 (p < 0.05, ANOVA) (**a**), AtHb2 (p < 0.01) (**b**), AtHb3 (p < 0.001) (**c**) bovine hemoglobin (p < 0.01) (**d**). At 8 µM Hb, in phosphate buffer, 100 mM, pH 7.

With the hypothesis that common intracellular electron donors will slow down/counteract the autoxidation, the effects of ascorbate, glutathione and NADH upon the oxy form of the three phytoglobins (and of bovHb as control) were investigated. The results are illustrated in Fig. 7 and indicate that with few exceptions these antioxidants *induce* autoxidation. NADH has the highest potential for promoting autoxidation. The latter observation may be explained in two ways. One explanation would involve direct electron transfer to oxygen for AtHb2, possibly facilitated by an easier access for NADH to the heme pocket. Direct reduction of histidine-ligated oxy hemoproteins has to date not been demonstrated in globins but is well-known in heme oxygenase (with electrons supplied by flavin) and in cytochrome *c* oxidase (with electrons supplied by nearby metallic centers)^83^. An alternative explanation would be an NADH-induced conformational change that leads to increased solvent exposure and hence increased autoxidation.

AtHb1 appears to be the most resistant to autoxidation at the studied concentrations, possibly also because NADH is an excellent reducer for metAtHb1^31^. In the case of AtHb1 and AtHb3, glutathione does not have any notable effect upon autoxidation, neither acceleration nor inhibition, especially if below 5 mM. Ascorbate accelerates autoxidation for all types of hemoglobin – for AtHb1 and AtHb2 only at high concentration, while for AtHb3 the effect is very pronounced even at the lowest concentration studied. All three antioxidants promote autoxidation of bovine hemoglobin very efficiently.

### Reduction reactions

The assumption that some of the plant antioxidants may be involved in the reduction of met hemoglobin was verified by incubating ferric nsHbs with various concentrations of ascorbate, glutathione and NADH; high concentrations were employed so as to more clearly observe the reaction. The corresponding increase in absorbance along with reduction of Fe^3+^ was measured for all three nsHbs (Fig. S9). AtHb2 could only be reduced by ascorbate, whileAtHb1 was reduced by all three compounds, but with different rates – and NADH appears as best reducing agent for AtHb1. In this latter case, a lag phase was observed. AtHb3 is also quite sensitive to all the studied reductants; however, an increase of the concentration of NADH and glutathione induces protein destruction. At higher concentrations, ascorbate reduces bovine methemoglobin faster and by a different mechanism that implies generating oxyhemoglobin within 10 minutes – which is further transformed to deoxyhemoglobin and eventually leads to heme degradation. At a very high concentration, ascorbate can undergo comproportionation leading to ascorbyl radical - which can oxidize the iron ion to higher valence states^84^. In the presence of O_2_, the hydroxyl radical can be generated^85^ – leading to ferryl formation, too. Because of the very high concentrations required for methemoglobin reduction in these experiments, one may conclude that these plant globins do not employ ascorbate, NADH or glutathione as sole direct reducing partners *in vivo* but rather that an enzymatic route would be expected. Our preliminary data suggest that *Arabidopsis* leaf extracts may contain an nsHb reductase activity (i.e., an analogue of the vertebrate methemoglobin reductase) based on metHb reduction exclusively in presence of plant extract and NADH but not in the presence of NADH alone. While we have at this time been unable to identify and characterize an enzyme responsible for catalyzing nsHbs reduction from leaf extracts, it may be worth to note that vertebrate Hbs are known to be reduced efficiently even if found extracellular – with membrane-bound cytochrome *b*_5_ enzymes from erythrocytes^29^.

To conclude, optical and Raman spectroscopy investigations of the three recombinant *Arabidopsis* phytoglobins in terms of their redox state and autoxidation reveal that their reactivity is highly dependent upon their coordination and heme binding site environment as well as upon their reaction partners. The more strongly hexacoordinated AtHb2 presents the highest autooxidation tendency whereas the pentacoordinated AtHb3 presents the lowest one. Autoxidation of AtHb2 in the presence of thiocyanate, chloride and fluoride suggests a nucleophilic displacement of superoxide, while for AtHb1 and AtHb3 this mechanism appears to be manifest only for thiocyanate. Oxyhemoglobin transformation to methemoglobin was accelerated by ascorbate and NADH for all proteins and by glutathione only for AtHb2; some of the tested antioxidants display a pro-oxidant effect at higher concentrations. These effects may be explained by conformational changes induced at the heme by these small molecules, possibly hinting at a physiological mechanism whereby the reactivity of nsHbs may be regulated conformationally. Alternatively, a less likely previously unexplored direct reduction of oxy heme by ascorbate/NADH/glutathione may be invoked.

## Electronic supplementary material

Supplementary information
