# Supplementary material for: Redox control and autoxidation of class 1, 2 and 3 phytoglobins from Arabidopsis thaliana
Source: Sci Rep. 2018 Sep 12;8:13714. doi: 10.1038/s41598-018-31922-4 (PMC6135765; doi:10.1038/s41598-018-31922-4)
Supplement: Supplementary file 1 — Supplementary information [file 41598_2018_31922_MOESM1_ESM.docx]

**Supplementary Information**

**Redox control and autoxidation of class 1, 2 and 3 phytoglobins from *Arabidopsis thaliana***

**Augustin C. Moț^1,2,3^*, Cristina Bischin^2^, Patricia Miclea^2^, Galaba Naumova Leția^2^, Sorin Dorneanu^2^, Dorina Podar^4^, Nico Dissmeyer^3^, Radu Silaghi-Dumitrescu^2^***

*^1^Research Center for Advanced Chemical Analysis, Instrumentation and Chemometrics, Babes-Bolyai University, 11 Arany Janos Street, RO-400028, Cluj-Napoca, Romania*

*^2^Faculty of Chemistry and Chemical Engineering, Babes-Bolyai University, 1 Mihail Kogalniceanu Street, RO-400084, Cluj-Napoca, Romania*

^3^Independent Junior Research Group on Protein Recognition and Degradation, Leibniz Institute of Plant Biochemistry, Weinberg 3, D-06120 Halle (Saale), Germany

*^4^Faculty of Biology and Geology, Babes-Bolyai University, 1 Mihail Kogalniceanu Street, RO-400084, Cluj-Napoca, Romania*

**emails:* [*augustinmot@chem.ubbcluj.ro*](mailto:augustinmot@chem.ubbcluj.ro)*,* [*rsilaghi@chem.ubbcluj.ro*](mailto:rsilaghi@chem.ubbcluj.ro)


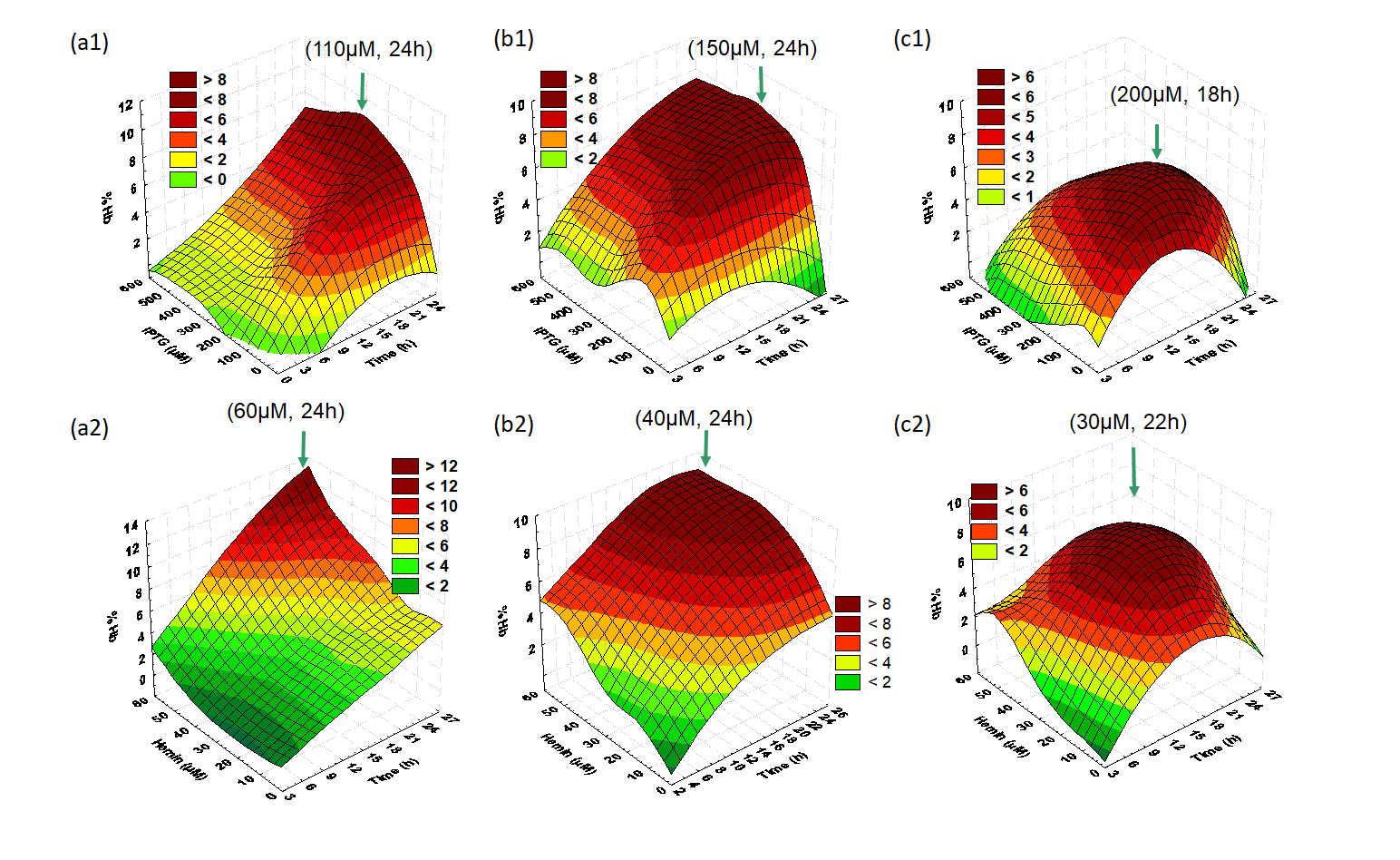


**Figure S1.** **Recombinant expression of phytoglobins in a partial factorial design.** Yields of non-symbiotic hemoglobins with varying concentrations of IPTG, heme and time of expression: AtHb1 (**a1,2**.), AtHb2 (**b1,2**.), AtHb3 (**c1,2**.).

**
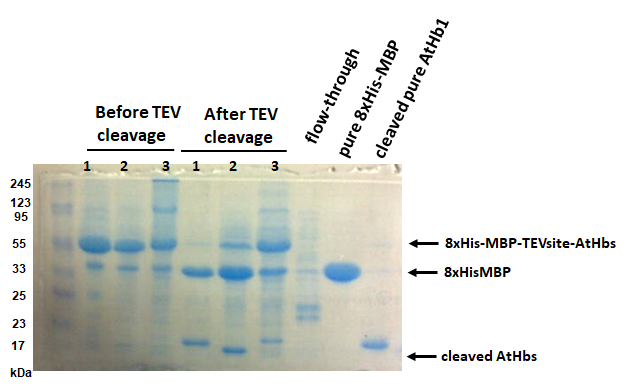
**

**Figure S2. Purification of recombinant phytoglobins.** SDS-PAGE electrophoresis before and after the cleavage of the affinity purification tag (His8-MBP) with TEV protease for AtHb1 (1), AtHb2 (2) and AtHb3 (3). Figure related to Figure 1.


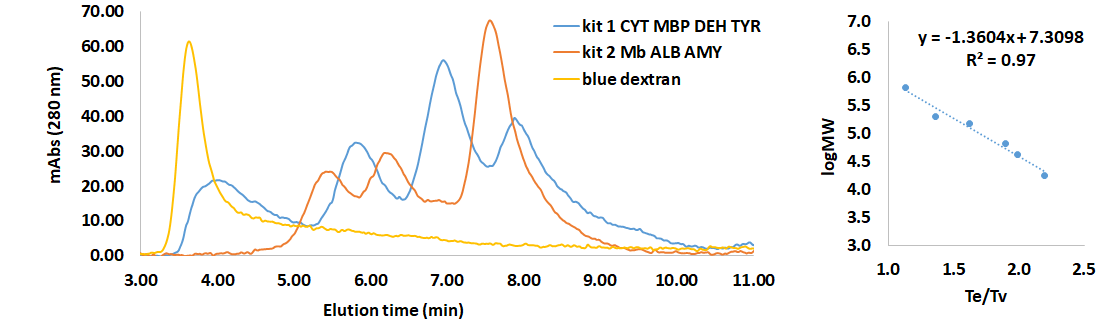


**Figure S3. Analytical size exclusion chromatography of recombinant phytoglobins.** Calibration kits and calibration curves used in determination of the apparent size of studied recombinant proteins (CYT – cytochrome c, MBP – maltose binding protein, DEH – alcohol dehydrogenase, TYR – thyroglobulin, Mb – myoglobin, ALB – albumin, AMY – amylase).

(a)


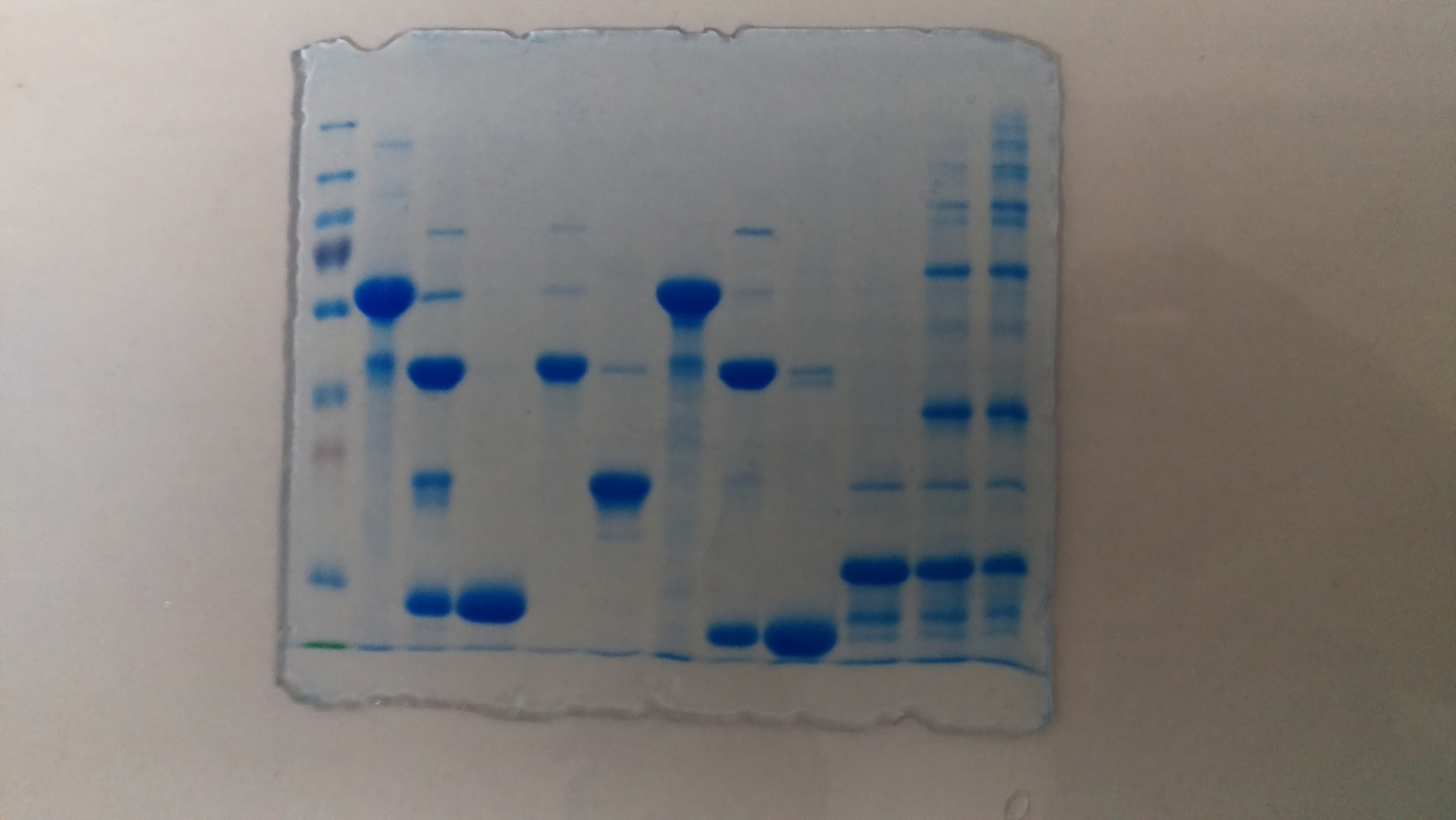

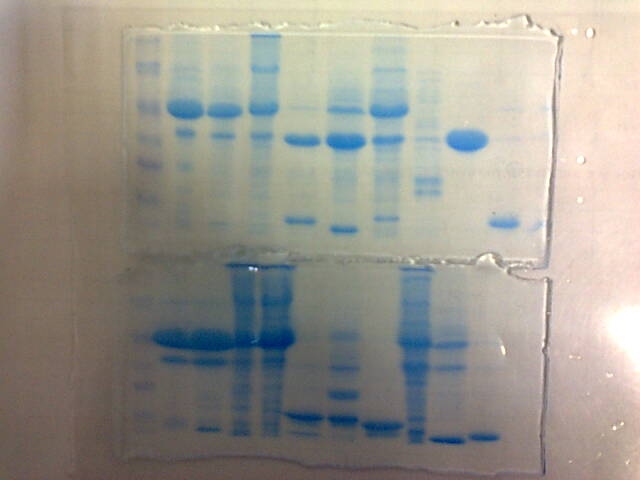


(b)

**Figure S4. As-photographed full length gels of the cropped parts presented in Figure 1 – main manuscript.** The cropped parts presented in Figure 1 are indicated in red rectangles. SDS-PAGE showing the TEV mediated His8-MBP removal for AtHb1 and AtHb2 (**A.**) and final anion exchange purification of AtHb3 (**B.**) as detailed in Figure 1 from main manuscript. Samples in the other lanes are related samples but without significant importance to be detailed. Figure related to Figure 1.


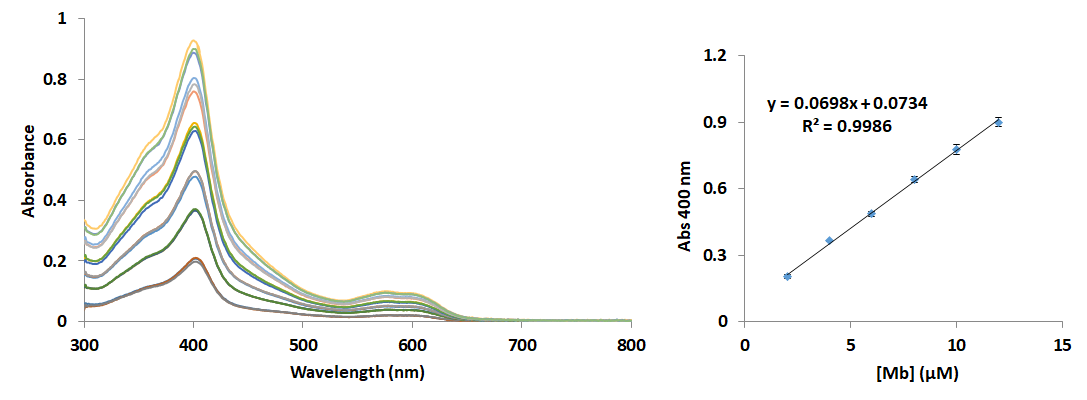


**Figure S5. Heme determination of recombinant phytoglobins by alkaline method.** Calibration curve used for heme determination of the three nsHbs using myoglobin as standard. Figure related to Table 1.

**
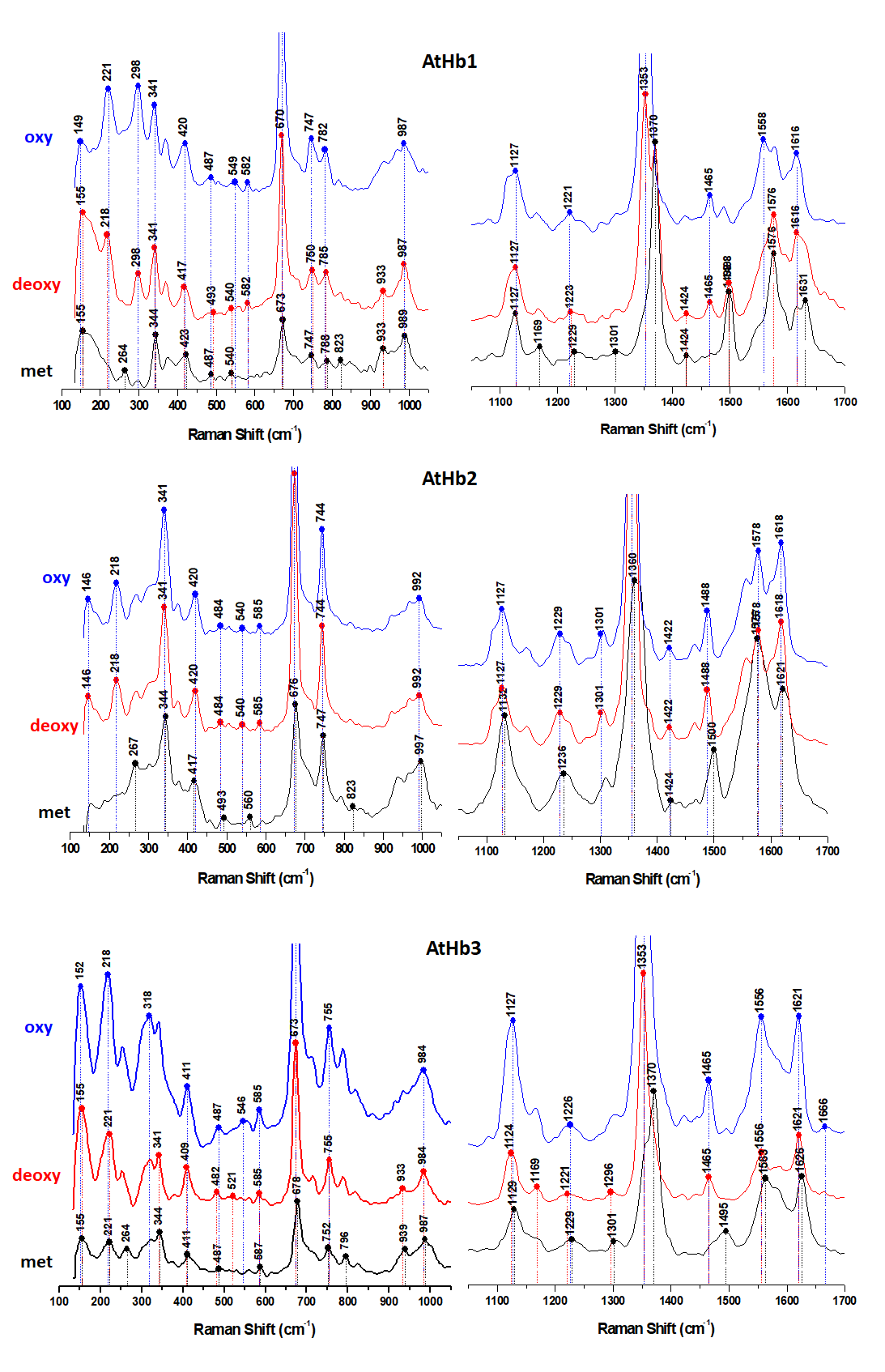
**

**Figure S6. Resonance Raman spectra of recombinant phytoglobins.** The three studied nsHbs were tested in the oxy, deoxy and met forms – low-frequency (left) and high-frequency (right) regions. The laser-excitation wavelength was 442 nm, and the proteins were in phosphate buffer pH 7.4. Figure related to Figure 3.

**
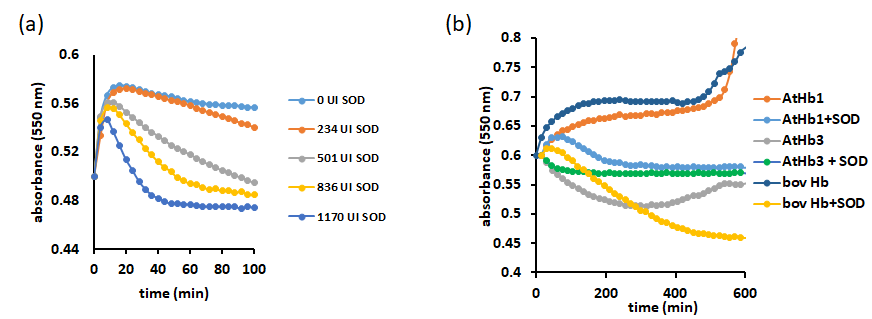
**

**Figure S7. Kinetic curves of recombinant phytoglobins autoxidation.** Kinetic curves of **A.)** cytochrome *c* reduction at 550 nm in the presence of, catalase and various concentrations of SOD and oxyAtHb2 and of **B.)** oxyAtHb1, AtHb3 or bovine hemoglobin, catalase and with or without SOD (376 UI). Figure related to Figure 6.


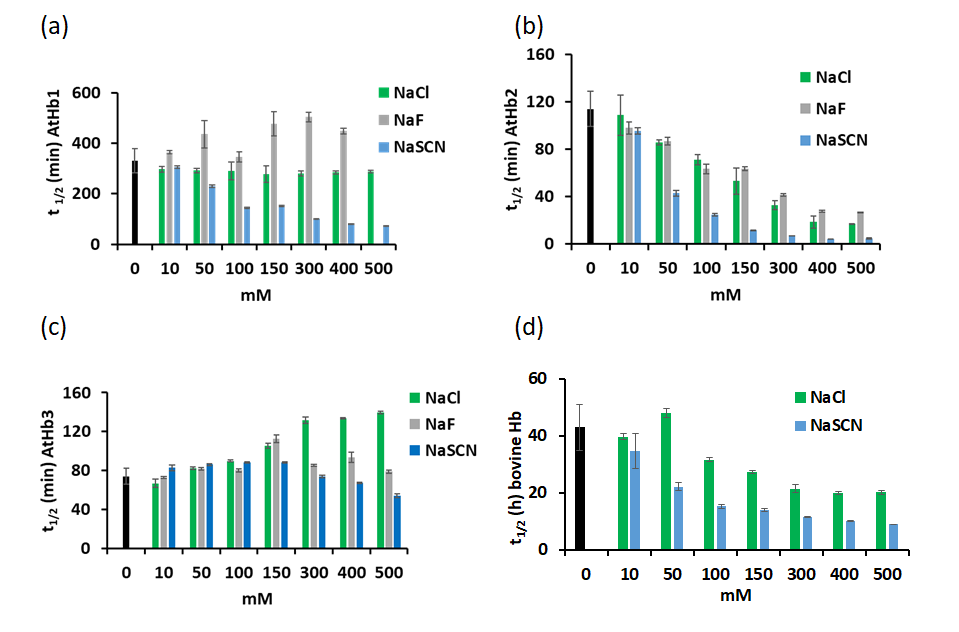


**Figure S8.** **Autoxidation of phytoglobins dependent upon anions concentrations.** Autoxidation (t_1/2_) in the presence of fluoride, chloride and thiocyanate for AtHb1 (p < 0.01, ANOVA) (**a**), AtHb2 (p < 0.01) (**b**), AtHb3 (p < 0.01) (**c**) and bovine hemoglobin (p < 0.01) (**d**). The reactions were performed in phosphate buffer, pH 7, at room temperature. The black bars represent the control sample: proteins without anions.


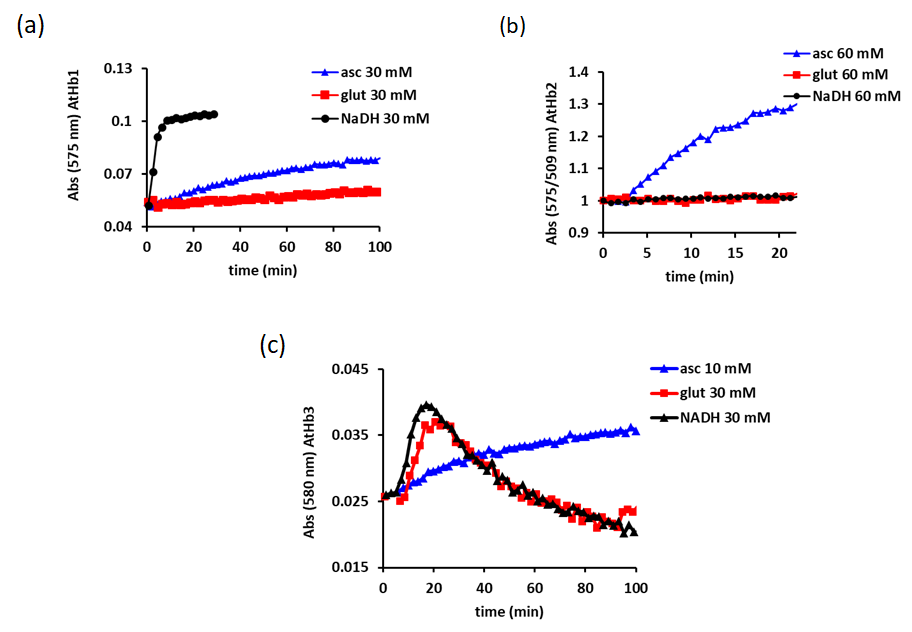


**Figure S9.** **Reduction of phytoglobins dependent upon reducing agents.** Reduction of metHb monitored by the specific growth in absorbance in the presence of ascorbate (asc), glutathione (glut) and NADH of AtHb1(**a**), AtHb2 (**b**) and AtHb3 (**c**). The protein was used at a concentration of 8 µM, in phosphate 100 mM, pH 7.
